# Silicone Implant Coated with Tranilast-Loaded Polymer in a Pattern for Fibrosis Suppression

**DOI:** 10.3390/polym11020223

**Published:** 2019-01-30

**Authors:** Byung Hwi Kim, Beom Kang Huh, Won Suk Lee, Cho Rim Kim, Kyu Sang Lee, Sun-Young Nam, Miji Lee, Chan Yeong Heo, Young Bin Choy

**Affiliations:** 1 Department of Biomedical Engineering, College of Medicine, Seoul National University, Seoul 03080, Korea; lierline@naver.com; 2 Interdisciplinary Program for Bioengineering, College of Engineering, Seoul National University, Seoul 08826, Korea; bkhuh85@snu.ac.kr (B.K.H.); siklight@naver.com (W.S.L.); chorim721@snu.ac.kr (C.R.K.); 3 Department of Pathology, Seoul National University Bundang Hospital, Seongnam 13620, Korea; tiqerkyu@gmail.com (K.S.L.); 4 Department of Plastic and Reconstructive Surgery, Seoul National University Bundang Hospital, Seongnam 13620, Korea; 99261@snubh.org (S.-Y.N.); r2404@snubh.org (M.L.); 5 Department of Plastic and Reconstructive Surgery, College of Medicine, Seoul National University, Seoul 03080, Korea; 6 Institute of Medical & Biological Engineering, Medical Research Center, Seoul National University, Seoul 03080, Korea

**Keywords:** drug delivery, fibrosis, pattern coatings, silicone implant, tranilast

## Abstract

Pathologic fibrosis around silicone implants is problematic, and thus, these implants have been coated with a mixture of a biocompatible polymer and antifibrotic drug for sustained drug release to prevent fibrosis. However, a coating applied over an entire surface would be subject to mechanical instability as the implant would be severely crumpled for implant insertion. Therefore, in this work, we proposed localized, patterned coating dots, each composed of poly(lactic-*co*-glycolic acid) (PLGA) and tranilast, to be applied on the surface of silicone implants. The drug loaded in the pattern-coated implant herein was well retained after a cyclic tensile test. Due to the presence of PLGA in each coating dot, the tranilast could be released in a sustained manner for more than 14 days. When implanted in a subcutaneous pocket in living rats for 12 weeks, compared with the intact implant, the pattern-coated implant showed a decreased capsule thickness and collagen density, as well as less transforming growth factor-β (TGF-β) expression and fewer fibroblasts; importantly, these changes were similar between the surfaces with and without the coating dots. Therefore, we conclude that the pattern-coating strategy proposed in this study can still effectively prevent fibrosis by maintaining the physical stability of the coatings.

## 1. Introduction

There has been a gradual increase in breast reconstruction surgeries due to the increase in breast cancer, as well as increased interest in aesthetic breast augmentation [1,2,3,4]. Various methods are available for breast reconstruction, including implantation of silicone implants, which is one of the most commonly used procedures [5,6,7]. Nondegradable and relatively inert silicone implants can secure a needed volume almost permanently and provide patients with aesthetic satisfaction [8,9,10]. Although widely accepted in clinical settings, capsular contracture is reported to be one of the major side effects and is caused by pathologic fibrosis around silicone implants [11,12].

Fibrosis is mainly caused by the foreign body reaction, where a nondegradable, bulky implant leads to prolonged inflammation that progresses towards a chronic stage, where excessive collagenous tissues are deposited around silicone implants [13,14]. During this process, a proinflammatory cytokine, transforming growth factor-β (TGF-β), is known to be overexpressed to recruit a large number of fibroblasts and produce excessive collagen [15,16,17]. Therefore, to prevent fibrosis, tranilast, a TGF-β-suppressing drug, is often prescribed for oral administration for a period of at least 90 days after implant insertion in clinical settings [18]. However, long-term systemic exposure to tranilast for more than 30 days may increase the chance of adverse effects, such as liver damage, anemia, and renal failure [19].

In this respect, silicone implants enabled with local delivery of tranilast at the site of implantation could be advantageous: Local exposure to a low-dose drug would still be effective without unnecessary systemic exposure. In addition, considering the prolonged period of inflammation, sustained drug release could benefit from a more pronounced antifibrotic effect [20,21]. For this purpose, in our previous study, the surface of implants was coated with a mixture of the drug and a biocompatible polymer, poly(lactic-*co*-glycolic acid) (PLGA) [22], which could indeed decrease fibrosis around silicone implants. It has been reported that PLGA could work as a diffusion mediator, hence allowing local, sustained release of drugs [23]. However, in practice, a coating around the entire surface of implants may not be physically stable. As the incision wound made for implantation should be minimized [24,25,26], a silicone implant is folded and crumpled for insertion, and thus, a coating on the entire surface would undergo severe mechanical stress with a high chance of breakage or loss [27].

Therefore, we propose patterned coatings on the surface of silicone implants for local, sustained delivery of tranilast. In this work, instead of coating the entire surface, we prepared coating dots composed of PLGA and tranilast, with each dot separated to cover only a selected area of the implant surface. In this way, we aimed to retain the physical stability of the coatings on the implant surface even after the severe mechanical stress often encountered during implant insertion through a minimized surgical incision. After implantation, we also aimed for the drug to be released in a sustained manner from each of the coating dots and aimed for the drug to diffuse towards the areas without the dots, hence becoming effective on the entire surface.

To assess the feasibility of this approach, we utilized the shell of a clinically approved silicone implant and pattern-coated the shell with several distinct dots composed of a mixture PLGA and tranilast. The pattern-coated implant was then subjected to mechanical stress mimicking the evaluation protocol needed for its approval in clinical use [28,29], and the patterned coatings were assessed and compared with the coating applied over the entire implant surface. To evaluate the antifibrotic effect, the pattern-coated implant was inserted in the subcutaneous space of living animals, and the tissues around the implant were biopsied and analyzed at scheduled times for 12 weeks.

## 2. Materials and Methods

### 2.1. Materials

The silicone implant shells in clinical use (SFS-LP) were donated by Hans Biomed (Seoul, Korea). Tranilast (assay value ≥ 98%) was purchased from JW Pharmaceutical (Seoul, Korea). We purchased PLGA (50:50; inherent viscosity = 0.41 dL/g) from Lakeshore Biomaterials (Birmingham, AL, USA), and acquired dimethylformamide (DMF) and acetonitrile (ACN) from JT Baker (Phillipsburg, NJ, USA). A medical epoxy (EPO-TEK^®^ 301-2) was purchased from Epoxy Technology (Billerica, MA, USA). Isoflurane used for respiratory anesthesia was purchased from Hana Pharmaceutical (Seoul, Korea). Paraformaldehyde (4%) and an ammonia solution (28%–30%) were obtained from KCFC (Ansan, Korea) and Junsie Chemical (Tokyo, Japan), respectively. Xylene, ethanol, acetic acid (1%), and hydrochloric acid (35%–37%) were purchased from Duksan Pure Chemicals (Ansan, Korea). Modified Mayer’s hematoxylin and eosin (H&E) Y solutions were acquired from Richard-Allan Scientific (San Diego, CA, USA). Biebrich scarlet-acid fuchsin, phosphomolybdic acid, phosphotungstic acid, and aniline blue solutions were purchased from Sigma-Aldrich (St. Louis, MO, USA). Target-retrieval solution (10X) and antibody diluent were obtained from Dako (Jena, Denmark). Anti-TGF-β (ab92486) and anti-vimentin (ab92547) antibodies were purchased from Abcam (Cambridge, MA, USA). For 4′,6-diamidino-2-phenylindole (DAPI) staining, VECTASHIELD mounting medium with DAPI was purchased from Vector Laboratories (Burlingame, CA, USA).

### 2.2. Preparation of Implant Samples

**Figure 1:**
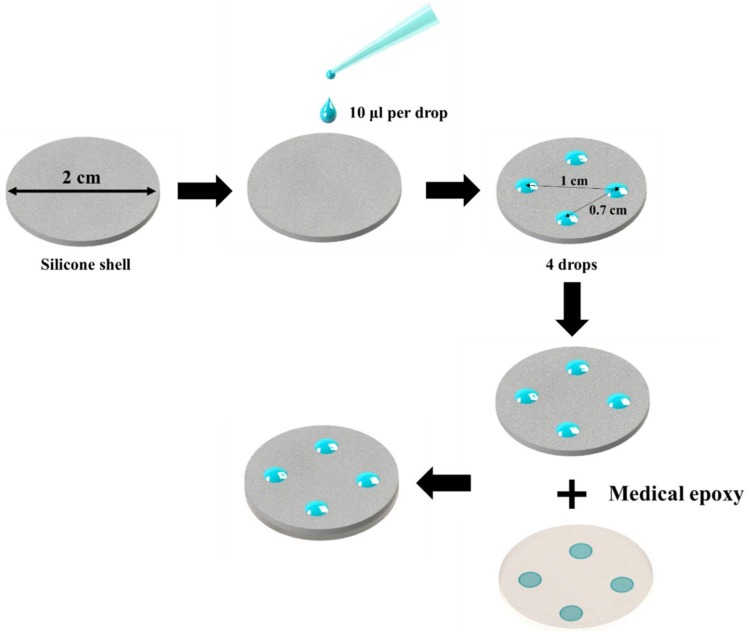
Schematic of the preparation of the pattern coatings on the surface of the implant shell.

In this study, we prepared the following three distinct samples using a shell of a silicone implant in a clinical setting: Intact implants (I/I: an intact shell without coatings), implants pattern-coated with PLGA only (PC/I), and implants pattern-coated with PLGA loaded with tranilast (PC/Tr/I). We also prepared implants coated over the entire surface with PLGA loaded with tranilast (EC/Tr/I) just to compare the physical stability of the coatings with that of the pattern-coated samples. To prepare the I/I, the implant shell was cut into circular pieces with a diameter of 2 cm, and two of those pieces were bonded together using a medical epoxy (EPO-TEK^®^ 301-2) with their inner surfaces facing each other. In this way, only the outer surface of the shell was exposed. To prepare the PC/I and PC/Tr/I, a solution of PLGA only (2% *w*/*v*) or a solution containing both PLGA (2% *w*/*v*) and tranilast (10% *w*/*w*) was prepared in DMF, respectively. Then, a drop of the resulting solution (10 µL/drop) was placed at each of the four locations on the outer surface of a circular implant piece to prepare the dotted coating patterns, as depicted in Figure 1. To prepare the EC/Tr/I, 200 µL of a solution containing both PLGA (2% *w*/*v*) and tranilast (10% *w*/*w*) in DMF was placed at the center on the outer surface of a circular implant piece to produce a hemispherical droplet covering the entire surface (Supplementary Figure S1). The coated pieces were then dried under vacuum for 24 h, and two of these pieces were then bonded as described above.

### 2.3. Sample Characterizations

The surface morphology of the silicone implants was analyzed using a scanning electron microscope (SEM; 7510F, JEOL, Tokyo, Japan). To measure the drug loading amount, the PC/Tr/I and EC/Tr/I were each immersed in 10 mL of DMF at room temperature for 1 h under sonication (NXPC-1505, Kodo Technical Research, Hwaseong, Korea) to fully dissolve the coating. Then, the solution was measured at a wavelength of 332 nm using a spectrophotometer (UV-1800, Shimadzu, Kyoto, Japan). To examine the physical stability of the coatings, the PC/Tr/I and EC/Tr/I underwent five cycles of tensile tests, using a Universal Testing Machine (UTM; Instron-5543, Instron, Norwood, MA, USA): The sample was elongated at a maximum strain of 450% and released to the initial length during a single cycle, which was repeated five times [28], and the changes in the surface morphology and drug loading amount were then assessed again as described above [29]. The surface of the implant samples herein was also assessed via attenuated total reflectance-Fourier transform infrared (ATR-FTIR) spectroscopy (TENSOR27, Bruker, Billerica, MA, USA) at a range of 400–4000 cm^−1^. To measure any residual DMF, each of the three different PC/Tr/I after drying was fully immersed in distilled water for 24 h, which was then assessed via high-performance liquid chromatography (HPLC; Ultimate 3000; Thermo Dionex, Waltham, MA, USA), using an Inno C_18_ column (250 mm × 4.6 mm, 5 µm, Young Jin Biochrom, Seongnam, Gyeonggi, Korea) [30]. The UV detection was set at 220 nm, and the mobile phase was made of a mixture of phosphate buffered saline (PBS) at pH 6.5 and ACN (95:5, *v*/*v*). The injection volume and flow rate were 10 µL and 1 mL/min, respectively.

To examine the in vitro drug release profiles, the PC/Tr/I was immersed in 50 mL of PBS at pH 7.4 and incubated at 37 °C with 125 rpm agitation (SI-300, Jeio Tech, Seoul, Korea) for 14 days. At predetermined time points of 1, 3, 6, and 12 h, and 1, 3, 5, 7, 10, and 14 days, we collected 10 mL out of the release medium (i.e., the obtained medium), and the same volume of fresh PBS was added back to maintain a good sink condition. The obtained medium was measured spectrophotometrically (UV-1800 Shimadzu, Kyoto, Japan) at a wavelength of 335 nm. The experiment was performed three times for each sample type. The data were plotted in cumulative drug release percent using the following equation:

(1) $$Y_{n}=Y_{n-1}+\frac{\left(C_{n}\times 50\;(mL)-C_{n-1}\times 40\;(mL)\right)}{\mathrm{Initial}\;\mathrm{drug}\;\mathrm{loading}\;\mathrm{amount}\;(\mu g)}$$

*Y_n_*: Cumulative drug release percent at the *n*th sampling time; *Y_0_* = 0
*C_n_*: Drug concentration in the medium obtained at the *n*th sampling time (μg/mL); *C*_0_ = 0

### 2.4. In Vivo Experiments

#### 2.4.1. Animals

The in vivo experimental protocol was verified and approved by the Institutional Animal Care and Use Committee (IACUC) of Seoul National University Bundang Hospital (Approval #: BA1302-122/009-01), and all experiments were conducted according to the regulation of Guidelines for Ethical Conduct in the Care and Use of Animals. In this work, we employed 8-week-old male Sprague Dawley rats (200–250 g), which were housed in specific pathogen free conditions with a 12-h day/night cycle and ad libitum access to food and water.

We assigned the animals into three distinct groups according to the type of implanted samples: The I/I, PC/I, and PC/Tr/I groups. A total of 25 animals were assigned to each group. The EC/Tr/I was not evaluated for in vivo studies as it was prepared just to compare the physical stability of the coatings with that of the pattern-coated samples. We inserted the sample in a subcutaneous space following the previous protocol [22,31]. At predetermined time points of 1, 2, 4, 8, and 12 weeks after sample insertion, five animals were randomly selected from each group and sacrificed using carbon dioxide to biopsy the tissues around the implant sample. The obtained tissue was fixed with a 10% formalin solution and stored at 4 °C until analysis.

#### 2.4.2. Histological and Immunofluorescence Analyses

For histological and immunofluorescence analyses, a slide was prepared by cutting a paraffinized block of the tissue sample into 4 μm thick slices, which were rehydrated and deparaffinized for staining. For H&E staining, the slide was dipped with a hematoxylin solution for 10 min and washed with tap water. Afterward, the slide was treated with the solutions of 0.3% HCl, 70% ethanol, and 0.1% ammonium hydroxide sequentially and then dipped into an eosin Y solution and washed with tap water. Masson’s trichrome (MT) staining was also carried out to assess the collagen density in the tissue around the implant. For this purpose, the slide was treated with a Biebrich scarlet-acid fuchsin solution and washed with water. Then, the slide was again treated with solutions of phosphotungstic-phosphomolybdic acid and aniline blue and then rinsed with tap water. For analyses of the degree of TGF-β expression and number of fibroblasts, immunofluorescence (IF) staining was performed using anti-TGF-β and anti-vimentin rabbit antibodies with a dilution factor of 1:250. For this step, the slide was first treated in an antigen retriever solution, heated for 15 min, and then washed with pH 7.4 PBS. Afterward, a goat serum blocking solution was applied for 30 min, followed by washing with PBS. The resulting slide was treated with a primary antibody and then stored at 4 °C for one day. After washing with PBS, the slide was treated with a secondary antibody at room temperature for 1 h, washed with PBS and dried at room temperature. Staining with DAPI was also performed using Vectashield mounting medium.

The stained slides were then examined by a professional pathologist in a blinded manner. For the I/I group, five images were randomly selected at each time of biopsy per animal group. For the PC/I and PC/Tr/I groups, five images were randomly selected for the tissue over the implant surface with or without the coating dot, thereby a total of 10 images at each time of biopsy per animal group. To assess the fibrotic capsule thickness, the H&E-stained slide was observed with an optical microscope (Carl Zeiss, Oberkochen, Germany) at 50× magnification, where the capsule thickness was measured from the muscle layer to the surface adjacent to the implant [22,31]. To evaluate the collagen density, TGF-β, and fibroblasts, the tissues in the capsule were observed at a higher magnification (200×). For assessment of the collagen density, the area of blue-colored collagen was measured as a percentage of the whole area of the image of the MT-stained slide [32]. For evaluation of TGF-β and fibroblasts, the IF-stained image was observed with a fluorescence microscope (Imager A1; Carl Zeiss, Oberkochen, Germany), and the cells with double positive signals for DAPI and a corresponding antibody were semiquantitatively analyzed as follows: 0; None, 1; Mild, 2; Moderate, and 3; Severe [33].

### 2.5. Statistical Analysis

We performed a nonparametric Mann-Whitney U test for statistical analysis of the capsule thickness, collagen density, degree of TGF-β, and fibroblast number among the tested groups, and a Bonferroni correction was additionally performed for statistical verification. We compared the experimental data between the I/I group and four other groups from the tissues over the surfaces with and without the coating dots of the PC/I and PC/Tr/I groups, respectively. The tissues over the surfaces with and without the coating dots were also specifically compared for the PC/I and PC/Tr/I groups, respectively. For all statistical analyses, *p* < 0.05 was considered statistically significant.

## 3. Results and Discussion

### 3.1. Characterizations

**Figure 2:**
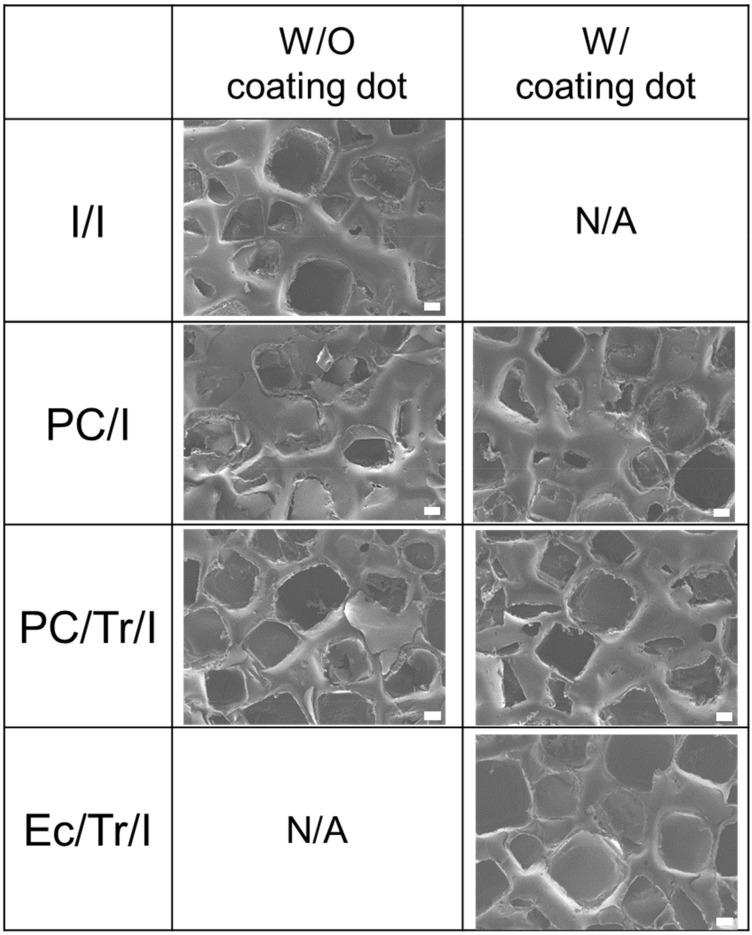
SEM images of the implant surfaces with coating dots (i.e., W/coating dot) and without the coating dots (i.e., W/O coating dot). Scale bars are 100 μm.

Figure 2 shows the SEM images of the implant surfaces, which exhibited square-shaped microwells due to the inherent textured surface of the implant shell used in this work (SFS-LP, Hans Biomed, Seoul, Korea). For the coated samples, the surface morphology was not very different between the surfaces with and without the coating dots, implying a thin layer of the coatings prepared in this work. For the PC/Tr/I, the drug loading amount was measured to be 284.39 ± 1.25 μg, which increased to 1389.07 ± 9.18 μg for the EC/Tr/I as more drug-loaded drops were utilized to coat the entire surface of the implant sample.

**Figure 3:**
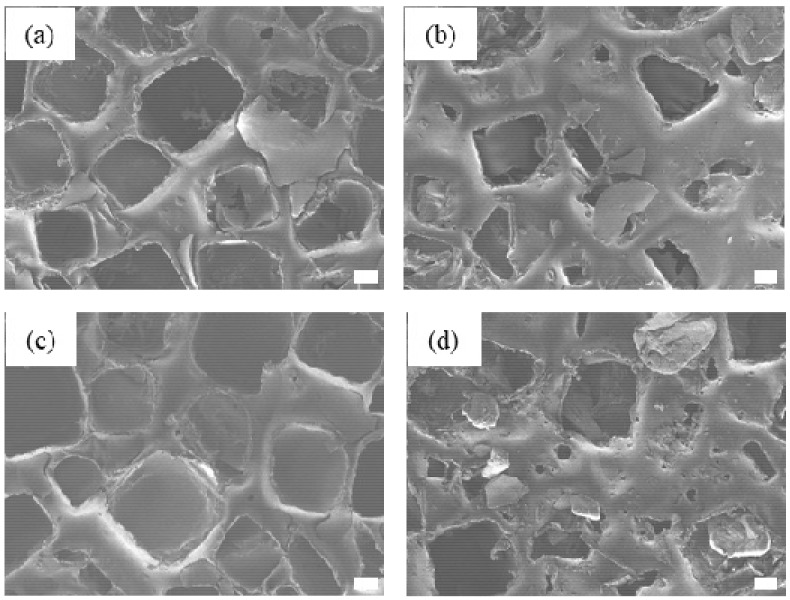
SEM images of the coatings before and after mechanical stress. The PC/Tr/I (**a**) before and (**b**) after mechanical stress and the EC/Tr/I (**c**) before and (**d**) after mechanical stress. Scale bars are 100 μm.

To examine the physical stability of the coatings, we assessed the implant samples actually loaded with tranilast. Thus, the PC/Tr/I and EC/Tr/I groups underwent cyclic mechanical stress (ASTM Standard F703) [29,30], and their surfaces were observed with a SEM. As shown in Figure 3, the coating damage was more apparent for the sample that was coated over the entire surface, i.e., the EC/Tr/I, than for the pattern-coated sample, i.e., the PC/Tr/I. Therefore, the decrease in the drug loading amount was also evident with the EC/Tr/I. As shown in Table 1, more than 20% drug loss was observed with the EC/Tr/I, while almost all the drug was retained with the PC/Tr/I (~99%). These results suggested that the coatings of the localized pattern could be well adhered to the silicone surface after mechanical stress, hence better stability during implant insertion.

**Figure 4:**
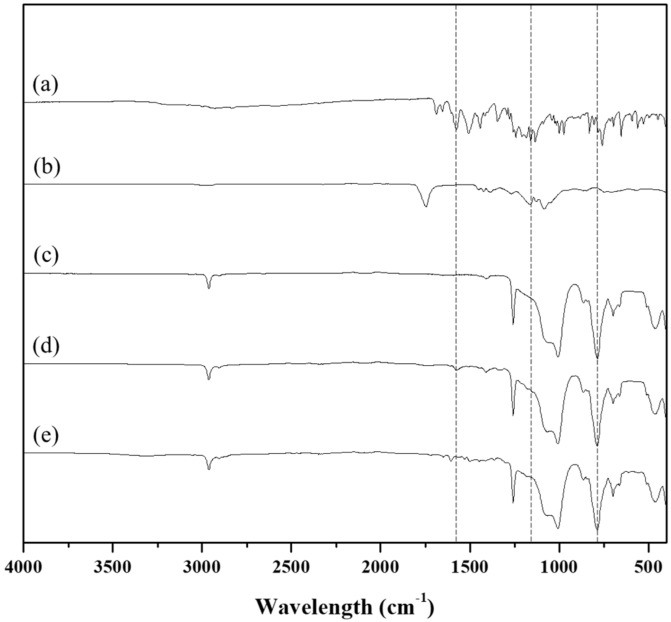
ATR-FTIR spectra of (**a**) intact tranilast, (**b**) intact PLGA, (**c**) I/I, and the surfaces of the PC/Tr/I (**d**) with and (**e**) without the coating dot. The dashed lines show the characteristic peaks.

Figure 4 shows the results from ATR- FTIR analysis. For intact tranilast, the characteristic peak was observed near the wavelength of 1500 cm^−1^ due to an ortho-disubstituted benzene group [22] and 1654 cm^−1^ due to amide C=O stretch [34]. For intact PLGA, a band appeared near at 1746, 1186 and 1084 cm^−1^, which could be ascribed to C=O, C–O–C, and C–O stretching, respectively [35,36,37]. The surface of the I/I showed a strong peak originating from silicone at 790, 1017 and 1260 cm^−1^ due to Si–(CH_3_)_2_, Si–O–Si, and C–H in Si–CH_3_, respectively [22,38,39]. For the PC/Tr/I, the surface with the coating dot exhibited the characteristic peaks that were overlapped with those from all constituent materials of PLGA, tranilast, and silicone while the one without the dot showed the characteristic peaks from the silicone only, as observed with the I/I. The PC/Tr/I herein was shown to contain a minimal amount of residual DMF (319.5 ± 7.0 ppm), which was less than its maximum concentration limit (880 ppm) allowed in the guideline of pharmaceuticals for human use [40].

**Figure 5:**
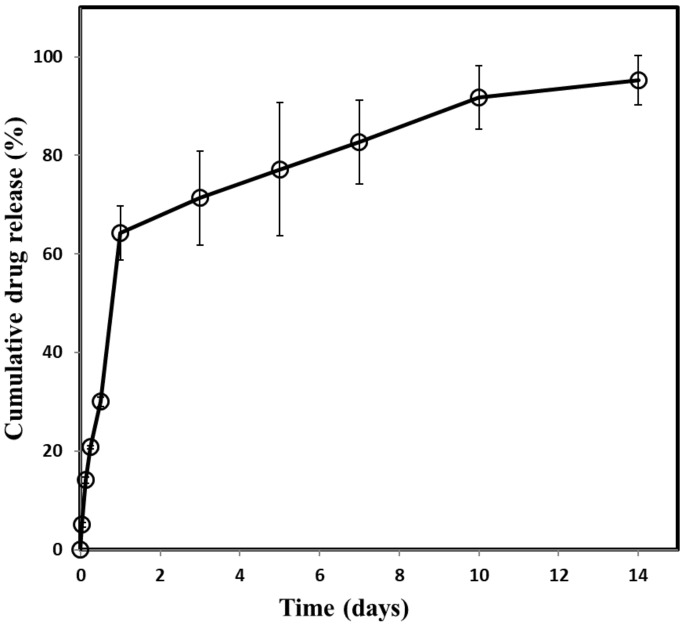
In vitro release profiles of tranilast from the PC/Tr/I. Error bars are the standard deviation.

We performed an in vitro drug release study with the PC/Tr/I, as shown in Figure 5. There was an apparent initial burst release of 60.15% ± 7.5% on the first day, which could be due to the higher drug distribution near the surface of PLGA coatings [41,42,43]. Afterwards, the drug was released in a sustained manner at a rate of approximately 2.56%/day until 14 days, indicating diffusion-mediated release of the drug encased in each of the coating dots of the PLGA matrix. When tested with fibroblast cells, the coating dots prepared in this work did not exhibit a statistically significant difference in cell adhesion compared with the intact silicone surface of the implant in clinical use (Figure S2).

### 3.2. In Vivo Evaluation

**Figure 6:**
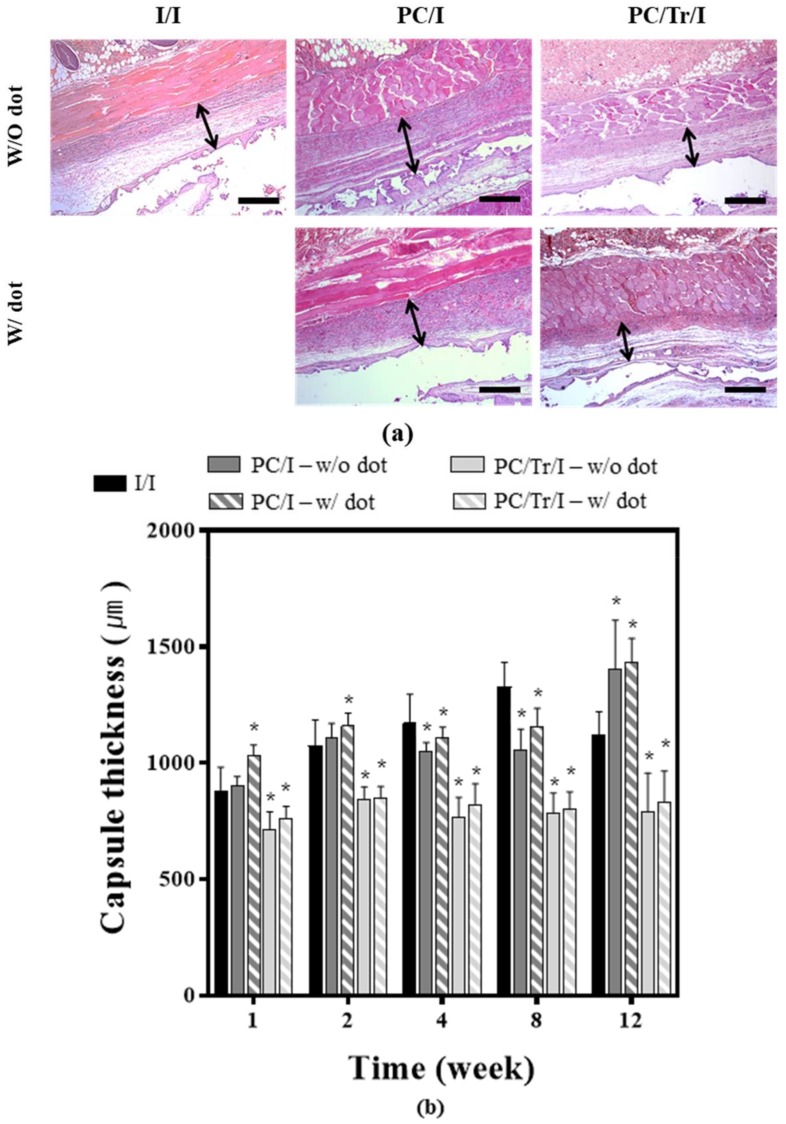
Evaluation of capsule thickness 12 weeks after sample implantation. (**a**) Representative H&E-stained tissue images observed at 12 weeks, where the double-ended black arrow indicates the capsule thickness. Scale bars are 1 mm. (**b**) Profiles of capsule thickness. * Statistically significant difference from the I/I (*p* < 0.05). Error bars are the standard deviation.

**Figure 7:**
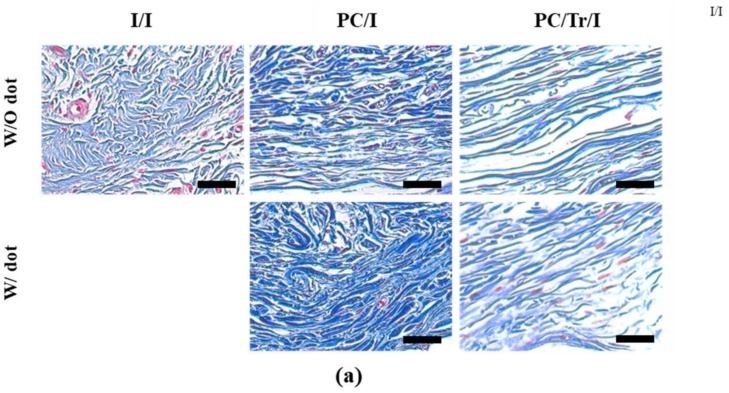

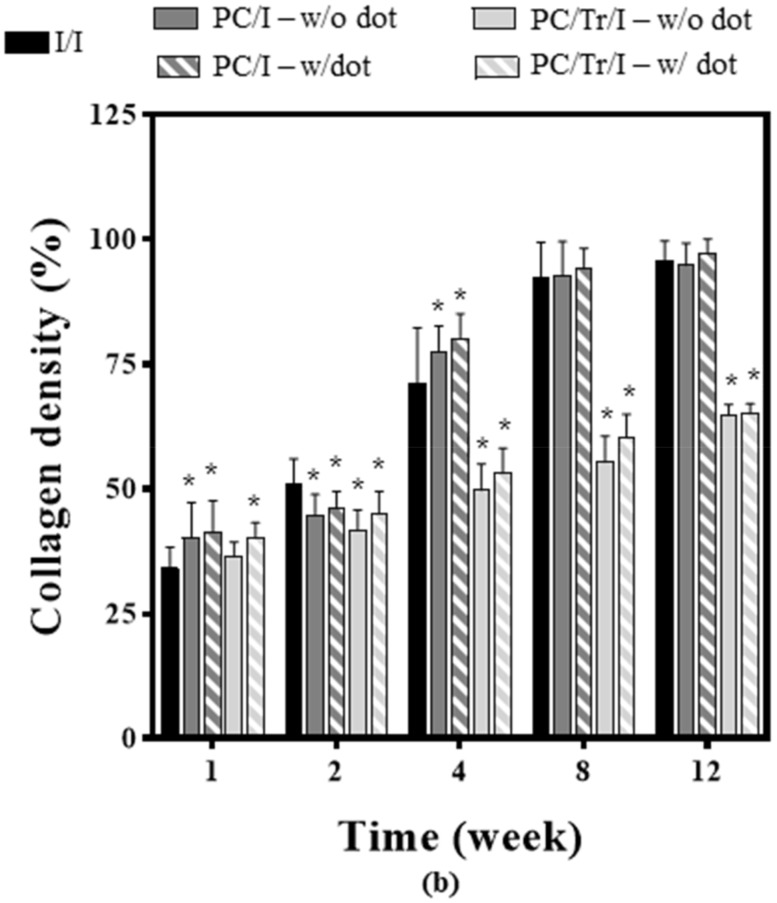
Evaluation of collagen density 12 weeks after sample implantation. (**a**) Representative MT-stained images observed at 12 weeks. Scale bars are 200 µm. (**b**) Profiles of collagen density. * Statistically significant difference from the I/I (p < 0.05). Error bars are the standard deviation.

To assess the antifibrotic efficacy of the implant samples, we first examined the capsule thickness of the H&E-stained tissues after sample implantation. As shown in Figure 6, the tranilast-loaded samples, i.e., the PC/Tr/I exhibited thinner capsules, which were significantly different from those of the samples without the drug, i.e., the I/I and PC/I, for the whole testing period of 12 weeks. The decrease in collagen density was also evident with the PC/Tr/I (Figure 7), which had a statistically significantly lower density than that of the I/I at 2–12 weeks. For the I/I and PC/I without tranilast, a gradual increase in collagen density was apparent, suggesting the formation of fibrotic tissues around the implanted silicone samples.

**Figure 8:**
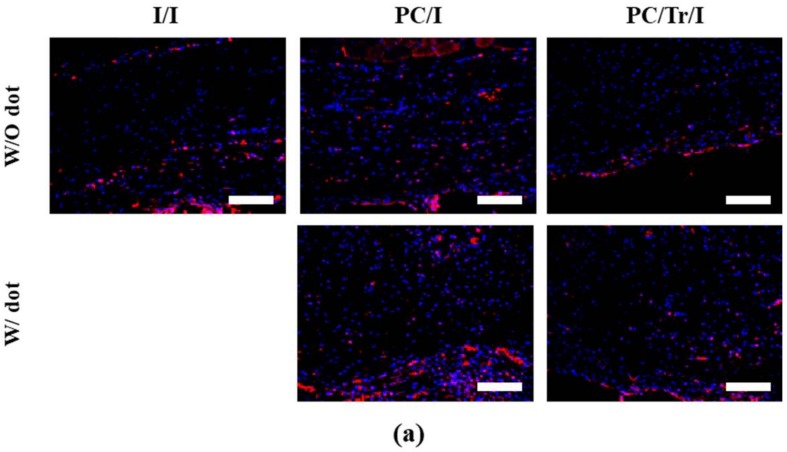

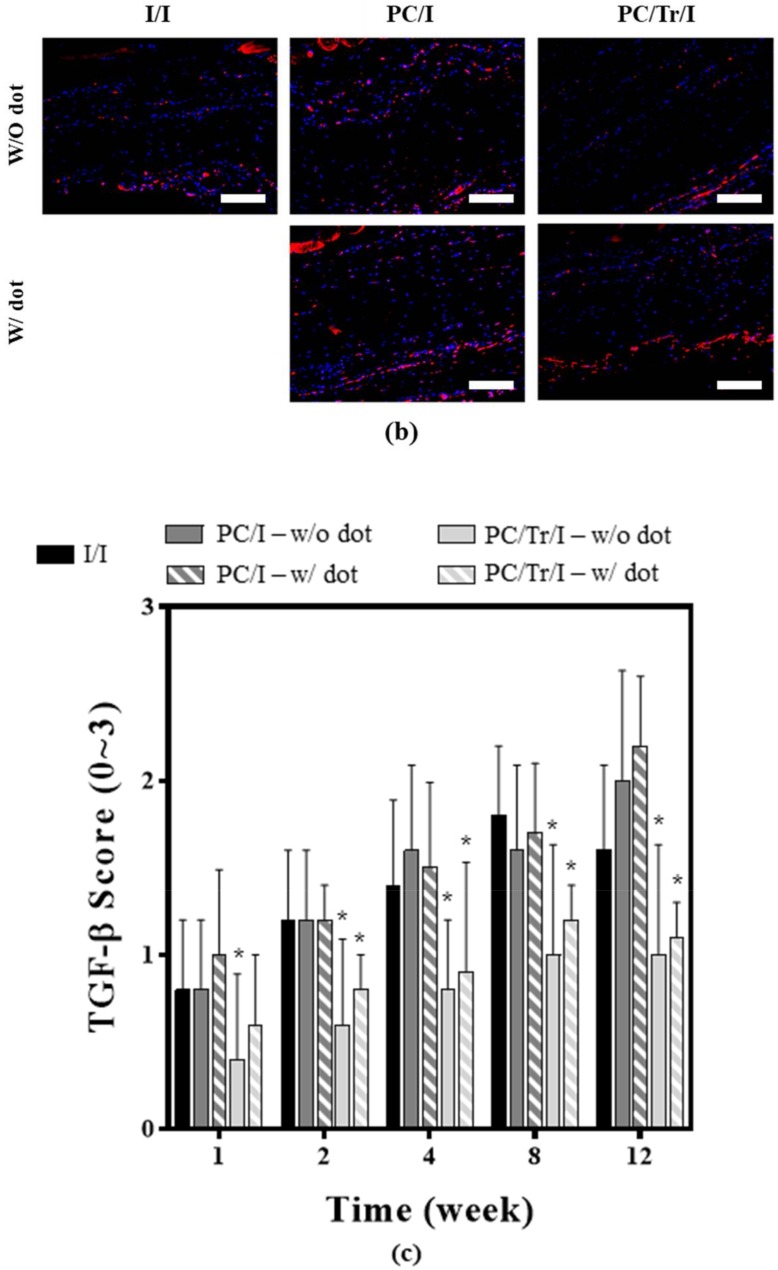
Evaluation of the TGF-β expression level 12 weeks after sample implantation. Representative IF-stained images of TGF-β expression at (**a**) 4 and (**b**) 12 weeks. Scale bars are 200 µm. (**c**) Score profiles of TGF-β expression. * Statistically significantly difference from the I/I (*p* < 0.05). Error bars are the standard deviation.

**Figure 9:**
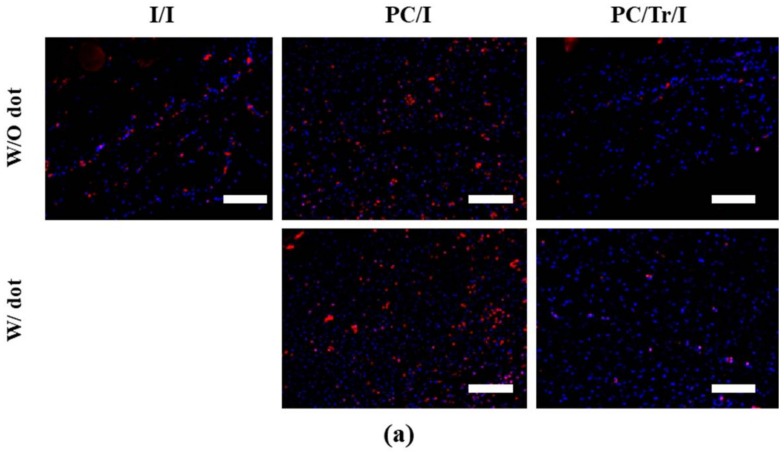

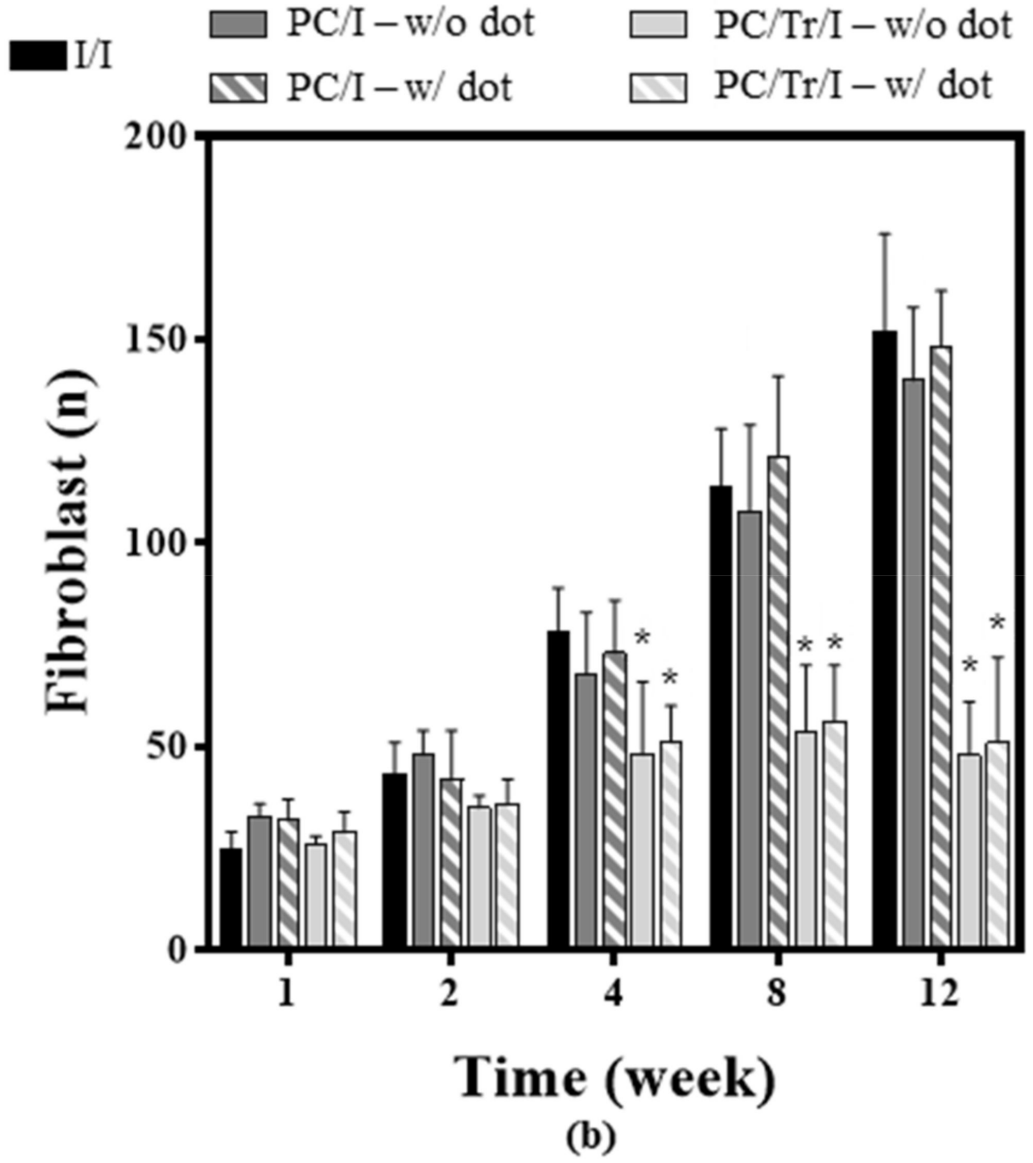
Evaluation of fibroblast number 12 weeks after sample implantation. (**a**) Representative IF-stained images of fibroblasts at 12 weeks. Scale bars are 200 µm. (**b**) Profiles of fibroblast number. * Statistically significant difference from the I/I (*p* < 0.05). Error bars are the standard deviation.

To further examine the efficacy of the drug, tranilast, we also sought to analyze the degree of TGF-β, as shown in Figure 8. The TGF-β expression was clearly lower in the PC/Tr/I than in the I/I and PC/I due to the presence of tranilast, a TGF-β inhibitory drug. Interestingly, this effect continued until 12 weeks, although the drug was almost depleted within two weeks (Figure 5). This finding could be explained by the cascading events from the acute to chronic stages of inflammation, as reported in our previous work [22]. Inhibition of TGF-β at the early stage of inflammation would decrease the number of monocytes, and thus, the macrophages that differentiated from monocytes would also decrease at the later stage of inflammation even without the presence of tranilast. During chronic inflammation, macrophages play a critical role in secreting TGF-β [44], and hence, a decreased number of macrophages could result in less TGF-β. Therefore, fewer fibroblasts were observed to be recruited with the PC/Tr/I, as shown in Figure 9. The number of fibroblasts was significantly lower for the PC/Tr/I than for the I/I and PC/I from 4 weeks. The lower number of fibroblasts, along with less TGF-β, appeared to cause less collagen synthesis (Figure 7) and hence smaller fibrotic capsules (Figure 6).

It should be noted that for the PC/Tr/I, all parameters evaluated under the present in vivo experiments were not significantly different between the tissues over the surfaces with and without the coating dots (Figure 6, Figure 7, Figure 8 and Figure 9). This result implied that the drug released from each of the coating dots spaced at the distance employed in this work could still cover and effectively reduce fibrotic tissue formation over the entire surface. All tested samples herein did not exhibit any apparent complications due to well-known biocompatibility of PLGA [45,46] and proven safety of the silicone implant used in this work (SFS-LP, Hans Biomed, Korea).

Silicone is known to possess a relatively low surface release energy [47,48] and thus, it would not be easy to retain the polymeric coating needed for sustained drug release, especially under a severe mechanical stress, inevitable during implant insertion. To improve the bonding property between the coated polymer and silicone, the silicone surface was often pre-treated before coating [49,50]. However, when the coating of a large area was made over the entire surface, it would still be susceptible to cracking during deformation of an implant itself, hence ease of breakage or loss of the coatings. Moreover, such an additional procedure may not be favored considering the efficiency in manufacturing. In this work, we prepared the patterned coating dots on the surface of a silicone implant in a relatively simple process. Thus, the coating dots exhibited a better physical stability as each dot would be less affected by the stress applied over a whole implant (Table 1 and Figure 3). When properly spaced, the coating dots in the implant could release the drug of interest to be effective over the entire surface, hence reducing fibrosis similarly effectively over both surfaces with and without the coating dots (Figure 6, Figure 7, Figure 8 and Figure 9).

## 4. Conclusions

We proposed patterned coating dots, each composed of a biocompatible polymer, PLGA, and tranilast, to be applied over the surface of a silicone implant to prevent pathologic fibrosis after implantation. Compared with a coating covering the entire surface, a patterned coating on the silicone implant could be more physically stable than a coating over the entire surface and thus, the patterned coating could retain the initial drug loading after experiencing the mechanical stress possibly associated with implant insertion. The pattern-coated silicone implant could release tranilast in a sustained manner due to the presence of a diffusion mediator, PLGA, in each of the coating dots. When implanted in living animals, the pattern-coated implant still reduced the capsule thickness and collagen density, with similar decreases for both surfaces with and without the coating dots. Therefore, we conclude that the preparation of drug-loaded, polymeric pattern coatings can be an advantageous approach for the preparation of a silicone implant capable of fibrosis suppression.

## Figures and Tables

**Table 1:** Drug loading amounts for the implant samples coated with tranilast-loaded PLGA.

Sample	Drug Loading Amount (µg/Sample)	
	Before Mechanical Stress	After Mechanical Stress
PC/Tr/I	284.39 ± 1.25	281.23 ± 5.48
EC/Tr/I	1389.07 ± 9.18	1105.09 ± 42.27

## Supplementary Materials

The following are available online at http://www.mdpi.com/2073-4360/11/2/223/s1, Figure S1: Schematic of the preparation of the entire coating on the surface of the implant shell. Figure S2: Cell adhesion property on the surfaces of the I/I, and the dots of the PC/I and PC/Tr/I, which was not statistically significantly different among the tested samples. For this analysis, we utilized the NIH 3T3 mouse embryo fibroblasts obtained from the American Type Culture Collection (Manassa, VA, USA). The cells were routinely grown in Dulbecco’s modified Eagle’s medium containing 1% antibiotic solution and 10% heat-inactivated fetal bovine serum at 37 °C in the presence of 5% CO_2_ and in a humidified atmosphere. The cells (3 × 10^5^ cells) were seeded on each of three different surfaces in a 24-well plate. After 1 h and 24 h incubation, cell adhesion was assessed by a MTT [3-(4,5-dimethylthiazol-2-yl)-2,5-diphenyltetrazolium bromide] assay following the manufacture’s instruction (Sigma-Aldrich, UK). In brief, 50 μL of 5 mg/mL MTT solution was added to each well containing 500 μL of the cell suspension and the cells were incubated at 37 °C for an additional 4 h. After removing the MTT solution, formazan crystals were dissolved in 1 mL of dimethyl sulfoxide (Sigma Chemical, St Louis, MO, USA) and the optical density was read using a microplate reader (BioTek, Winooski, Vermont, VT, USA) at a wavelength of 560 nm.
