# Supplementary material for: Silicone Implant Coated with Tranilast-Loaded Polymer in a Pattern for Fibrosis Suppression
Source: Polymers (Basel). 2019 Jan 30;11(2):223. doi: 10.3390/polym11020223 (PMC6419080; doi:10.3390/polym11020223)
Supplement: Supplementary file 1 [file polymers-11-00223-s001.pdf]

Supplementary Figure

Silicone Implant Coated with Tranilast-Loaded Polymer in a Pattern for Fibrosis Suppression

Byung Hwi Kim, Beom Kang Huh, Won Suk Lee, Cho Rim Kim, Kyu Sang Lee, Sun-Young Nam, Miji Lee, Chan Yeong Heo and Young Bin Choy

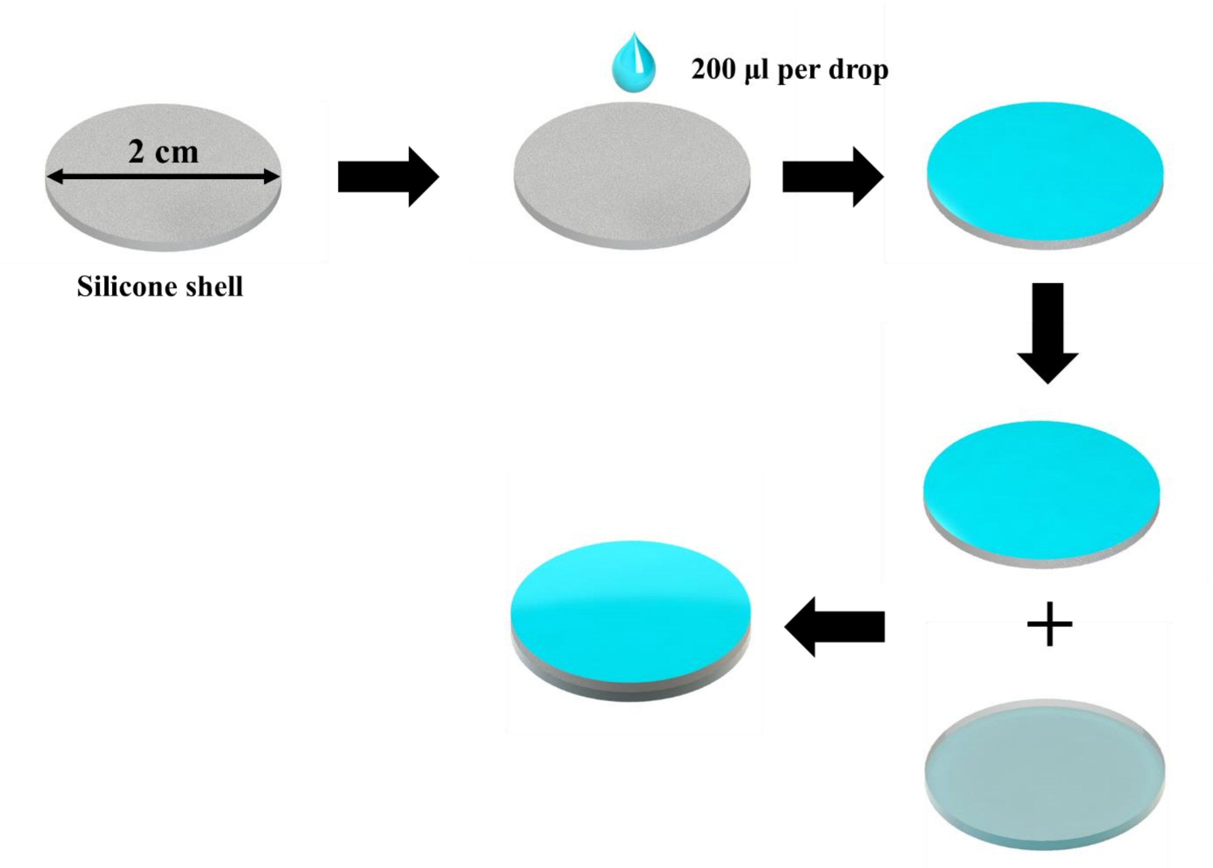

Figure S1. Schematic of the preparation of the entire coating on the surface of the implant shell.

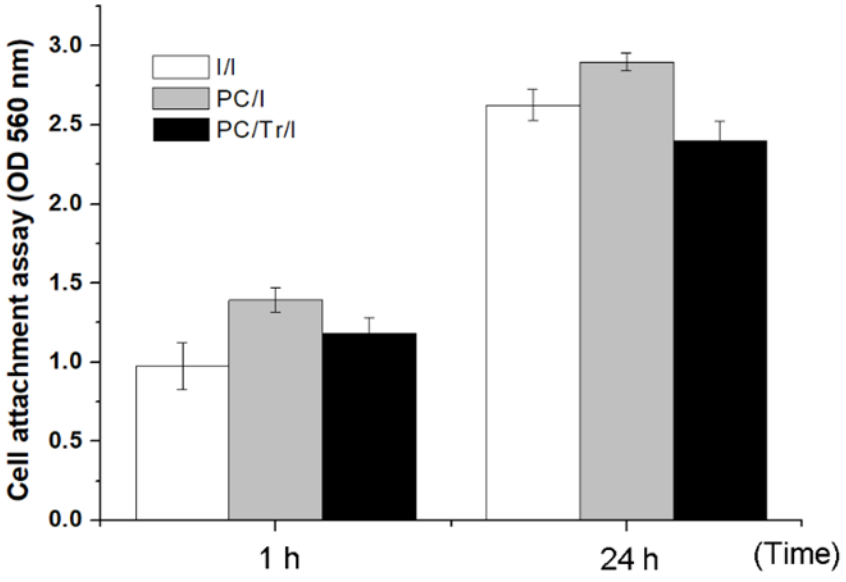

Figure S2. Cell adhesion property on the surfaces of the I/I, and the dots of the PC/I and PC/Tr/I, which was not statistically significantly different among the tested samples. For this analysis, we utilized the NIH 3T3 mouse embryo fibroblasts obtained from the American Type Culture Collection (Manassa, VA, USA). The cells were routinely grown in Dulbecco's modified Eagle's medium containing 1% antibiotic solution and 10% heat-inactivated fetal bovine serum at 37˚C in the presence of 5% CO_2 and in a humidified atmosphere. The cells (3×10^5 cells) were seeded on each of three different surfaces in a 24-well plate. After 1 h and 24 h incubation, cell adhesion was assessed by a MTT [3-(4,5-dimethylthiazol-2-yl)-2,5-diphenyltetrazolium bromide] assay following the manufacture's instruction (Sigma-Aldrich, UK). In brief, 50 μl of 5 mg/ml MTT solution was added to each well containing 500 μl of the cell suspension and the cells were incubated at 37˚C for an additional 4 h. After removing the MTT solution, formazan crystals were dissolved in 1 ml of dimethyl sulfoxide (Sigma Chemical, St Louis, MO, USA) and the optical density was read using a microplate reader (BioTek, Vermont, USA) at a wavelength of 560 nm.
